# The Combined Effect of Aging and Performance Level on Pacing in Duathlon – the “ITU Powerman Long Distance Duathlon World Championships”

**DOI:** 10.3389/fpsyg.2019.00296

**Published:** 2019-02-14

**Authors:** Pantelis T. Nikolaidis, Hamdi Chtourou, Rodrigo Ramirez-Campillo, Elias Villiger, Thomas Rosemann, Beat Knechtle

**Affiliations:** ^1^Faculty of Biomedical Sciences, University of East Attica, Egaleo, Greece; ^2^Exercise Physiology Laboratory, Nikaia, Greece; ^3^Institut Supérieur du Sport et de l’éducation Physique de Sfax, Université de Sfax, Sfax, Tunisia; ^4^Activité Physique: Sport et Santé, UR18JS01, Observatoire National du Sport, Tunis, Tunisia; ^5^Laboratory of Human Performance, Quality of Life and Wellness Research Group, Department of Physical Activity Sciences, Universidad de Los Lagos, Osorno, Chile; ^6^Institute of Primary Care, University of Zurich, Zurich, Switzerland; ^7^Medbase St. Gallen Am Vadianplatz, St. Gallen, Switzerland

**Keywords:** age group, cycling, endurance, master athletes, running, ultra-endurance

## Abstract

The role of age and performance level has been investigated in runners such as marathoners, but not in multi-sports athletes such as duathletes (running, cycling, and running). Thus, the aim of the present study was to examine the combined effects of aging and performance level on pacing of duathletes competing in two different race distances. Pacing (defined as the relative contribution of cycling time, %, to the overall race time) was analyzed for 6,671 duathletes competing from 2003 to 2017 in the short distance race (10 km first run, 50 km cycling and 5 km second run) or long distance race (10 km first run, 150 km cycling and 30 km second run) of “Powerman Zofingen,” the “ITU Powerman Long Distance Duathlon World Championships.” Men were faster, older, and spent less time (%) in cycling than women in both distances races (*p* < 0.001). Younger age groups spent more time (%) in cycling than their older counterparts in women (both short and long distance, *p* = 0.036, η_p_^2^ = 0.031, *p* = 0.025, η_p_^2^ = 0.044, respectively) and men (long distance race, *p* < 0.001, η_p_^2^ = 0.016). Fast performance groups spent more time (%) in cycling than their slower counterparts in short (women, *p* < 0.001, η_p_^2^ = 0.057; men, *p* < 0.001, η_p_^2^ = 0.035) and long distance (women, *p* < 0.001, η_p_^2^ = 0.070; men, *p* < 0.001, η_p_^2^ = 0.052). A small age group × performance group interaction on cycling time (%) was observed in the men’s short distance (*p* = 0.001, η_p_^2^ = 0.020) – but not in the long distance or in women – with smaller differences between performance groups in the older than in the younger age groups. Women, young and fast duathletes were relatively slower in cycling than men, old and slow duathletes; that was, old duathletes were relatively faster in cycling than in running. Moreover, there was indication that the difference in pacing among performance groups might be attenuated with aging. Since fast duathletes were relatively faster in running than in cycling, slow duathletes should be encouraged to cycle slower and run faster.

## Introduction

Pacing in endurance performance describes how an athlete invests energy during performance and the pacing strategy during a race can considerably influence the outcome of the race (Nikolaidis and Knechtle, 2017b). To date, six different pacing strategies have been identified such as negative, all-out, positive, even, parabolic-shaped and variable pacing strategies (Abbiss and Laursen, 2008). Pacing during endurance performance has been mainly investigated for runners (Diaz et al., 2018; Nikolaidis and Knechtle, 2018b), swimmers (Nikolaidis and Knechtle, 2017c; Rodriguez and Veiga, 2018), cyclists (Bossi et al., 2018; Granier et al., 2018) and multi-sports athletes such as triathletes (Angehrn et al., 2016; Knechtle and Nikolaidis, 2016). Regarding triathlon (i.e., swimming, cycling, running), pacing has been investigated for different distances such as sprint distance triathlon (Taylor and Smith, 2014; Wu et al., 2015, 2016), Olympic distance triathlon (Vleck et al., 2008), Half-Ironman triathlon (Wu et al., 2015), Ironman triathlon (Johnson et al., 2015; Angehrn et al., 2016), and longer triathlon distances (Herbst et al., 2011; Knechtle and Nikolaidis, 2016). The abovementioned studies focused on the variation of pacing by aspects such as sex, age and performance level and provided practical information for coaches in these sports in order to develop optimal pacing strategies for their athletes.

Apart from triathlon as a multi-sports discipline also duathlon (i.e., running, cycling, and running) exists. However, we have no knowledge how duathletes pace during races of different distances. Recent studies showed the importance of age in pacing. Indeed, studies investigating marathoners found that fast master runners (i.e., older than 35 years) pace differently than slow master runners (Nikolaidis and Knechtle, 2017a,b). Moreover, older runners with a similar race time pace differently than younger runners with smaller changes during the race (Nikolaidis and Knechtle, 2017b).

Duathlon is a multi-sports discipline with a first running leg, then a cycling leg, followed by a second running leg. This change in disciplines can dramatically change pace strategy to achieve the best performance or finish the race at all (Knechtle et al., 2015; Nikolaidis and Knechtle, 2017b). However, there is no evidence regarding pacing strategies in duathlon and about the possible effect of age on it. In addition, the existed knowledge about the role of sex, age and performance level on pacing in triathlon (Knechtle et al., 2019) could not be “transferred” to duathlon considering that these multi-sports differed for their disciplines. On the other hand, information on the role of these aspects on pacing in duathlon would be of practical interest for scientists and practitioners (e.g., coaches and fitness trainers) involving in this sport in order to design optimal pacing strategies considering whether they train women or men, young or older, elite or recreational duathletes. Therefore, the aim of the present study was to investigate the combined effects of aging and performance level on pacing of elite duathletes competing in two different race distances in the “ITU Powerman Long Distance Duathlon World Championships” held at “Powerman Zofingen” in Switzerland. Based upon previous findings for marathoners we hypothesized to find differences in the variation of pacing by performance between younger and older athletes, i.e., smaller differences in pacing among performance groups would be observed in the older age groups.

## Materials and Methods

### Ethics Statement

The Institutional Review Board of Kanton St. Gallen, Switzerland approved all procedures used in the study with a waiver of the requirement for informed consent of the participants given the fact that the study involved the analysis of publicly available data. The study was conducted in accordance with recognized ethical standards according to the Declaration of Helsinki adopted in 1964 and revised in 2013.

### Data

A total of 6,671 duathletes - for whom times for each duathlon’s discipline were available – competing from 2003 to 2017 in the “Powerman Zofingen” was considered in the present study. One case with missing age was excluded from further analysis resulting in a final sample of 6,670 duathletes (short distance, women, *n* = 556, men, *n* = 2945; long distance, women, *n* = 481, men, *n* = 2688). All analyses were performed based on data derived from the official race website www.powerman.ch of “Powerman Zofingen.” The “Powerman Zofingen” is a duathlon event held in Zofingen (Switzerland) within the “Powerman World Series.” In this race, a short and a long distance version are held. A duathlon consists of a running part, a cycling part and then again a running part, which are carried out directly after each other. Since 2002, the long distance race of “Powerman Zofingen” has the sequence of 10 km running, 150 km cycling and 30 km running. At the same time of the long distance race, also a short distance is held covering 10 km running, 50 km cycling, and 5 km running. Before 2003, the race course and the distances of the split disciplines changed several times since the first edition of the race in 1989.

### Procedures

Race results were sorted by name, age and sex of the finishers separately for both the short and the long distance race. Athletes were ranked in 5-year age groups from 15–19 years to 70–74 years and their distribution by sex, age and distance can be seen in Table 1.

**Table 1:** Cycling time (%) by sex, distance and age group.

	Short distance				Long distance			
Age group (years)	Women (*n* = 556)		Men (*n* = 2945)		Women (*n* = 481)		Men (*n* = 2688)	
	*n*	Cycling time (%)	*n*	Cycling time (%)	*n*	Cycling time (%)	*n*	Cycling time (%)
15–19	24	57.85 ± 2.12	107	57.51 ± 1.97	1	60.16	2	57.97 ± 2.23
20–24	49	58.04 ± 2.03	133	57.88 ± 2.50	7	59.63 ± 2.42	70	59.23 ± 2.33
25–29	100	58.41 ± 2.22	275	57.64 ± 2.36	68	60.50 ± 2.14	271	59.77 ± 2.20
30–34	108	57.53 ± 2.21	409	57.73 ± 2.12	103	60.27 ± 2.17	444	59.52 ± 2.17
35–39	99	57.95 ± 2.42	521	57.47 ± 2.01	89	60.19 ± 1.88	511	59.18 ± 2.17
40–44	89	57.48 ± 1.88	556	57.29 ± 2.00	76	59.50 ± 1.91	541	58.82 ± 2.12
45–49	56	57.33 ± 1.73	450	57.09 ± 1.93	76	59.24 ± 2.03	394	58.31 ± 2.12
50–54	25	57.94 ± 1.57	248	57.03 ± 1.82	42	57.82 ± 2.26	235	58.26 ± 2.46
55–59	6	58.63 ± 3.12	145	56.69 ± 1.74	14	56.61 ± 2.78	123	58.16 ± 2.32
60–64			77	55.86 ± 1.51	4	56.51 ± 1.19	59	56.81 ± 2.31
65–69			22	55.66 ± 2.13	1	56.67	32	57.07 ± 2.46
70–74			2	56.22 ± 0.71			6	54.78 ± 2.02


Performance (i.e., relative time) in cycling was expressed as percentage of overall race time using the formula “100 × disciplines” time/total time’ and was used as a measure of pacing. The relative time in disciplines were used previously in other multisport to study pacing (Knechtle and Nikolaidis, 2016). We examined the effect of age on pacing (i.e., defined as the relative contribution of cycling time, %, to the overall race time) of duathletes competing in the short distance race (i.e., 10 km first run, 50 km cycling and 5 km second run) or long distance race (i.e., 10 km first run, 150 km cycling, and 30 km second run).

Athletes were then also ranked in four quartile groups (Q1, Q2, Q3, and Q4) with Q1 the fastest and Q4 the slowest. In the short distance race, cut-offs for performance quartiles were Q1 ≤ 169.7 min, 169.7 < Q2 ≤ 183.4 min, 183.4 < Q3 ≤ 199.8 min, and Q4 > 199.8 min in women, and Q1 ≤ 157.3 min, 157.3 < Q2 ≤ 168.7 min, 168.7 < Q3 ≤ 183.7 min, and Q4 > 183.7 min in men. In the long distance race, cut-offs for performance quartiles were Q1 ≤ 498.8 min, 498.8 < Q2 ≤ 541.2 min, 541.2 < Q3 ≤ 584.1 min, and Q4 > 584.1 min in women, and Q1 ≤ 461.1 min, 461.1 < Q2 ≤ 499.6 min, 499.6 < Q3 ≤ 542.8 min, and Q4 > 542.8 min in men.

### Statistical Analysis

Data are presented as means ± standard deviations. A between-within measures analysis of variance (ANOVA) examined the distance × discipline interaction on relative time. Within each distance, a between-within measures ANOVA examined the sex × discipline and age group × discipline interaction on relative time. The magnitude of these interactions was examined using effect size partial eta square (η_p_^2^) and was evaluated as following: small (0.010 < η_p_^2^ ≤ 0.059), moderate (0.059 < η_p_^2^ ≤ 0.138) and large (η_p_^2^ > 0.138) (Cohen, 1988). Statistical analyses were carried out using GraphPad Prism v. 7.0 (GraphPad Software, San Diego, United States) and IBM SPSS v.23.0 (SPSS, Chicago, United States).

## Results

**FIGURE 1:**
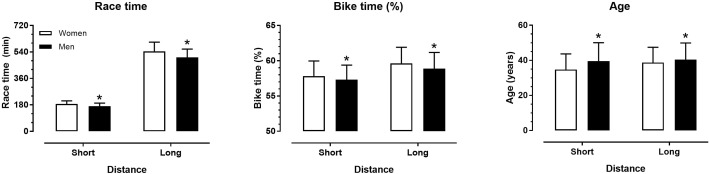
Race time, cycling time (%) and age by sex and distance. ^∗^different from women at *p* < 0.001.

Men were faster and older, and spent less time (%) in cycling than women in both distances (*p* < 0.001) (Figure 1). A small main effect of age on cycling time (%) was observed in women (both short and long distance, *p* = 0.036, η_p_^2^ = 0.031, *p* = 0.025, η_p_^2^ = 0.044, respectively) and men’s long distance (*p* < 0.001, η_p_^2^ = 0.016), whereas no effect was shown in men’s short distance (*p* = 0.092, η_p_^2^ = 0.006) (Table 1). According to this effect, athletes in younger age groups spent more time (%) in cycling than their older counterparts, i.e., young duathletes were relatively slow in cycling.

A small-to-moderate main effect of performance on cycling time (%) was found in the short distance (women, *p* < 0.001, η_p_^2^ = 0.057; men, *p* < 0.001, η_p_^2^ = 0.035) and the long distance race (women, *p* < 0.001, η_p_^2^ = 0.070; men, *p* < 0.001, η_p_^2^ = 0.052) (Table 2). Athletes in fast performance groups spent more time (%) in cycling than their slower counterparts, i.e., fast duathletes were relatively slow in cycling.

**Table 2:** Cycling time (%) spent in disciplines and transitions by distance.

	Short distance		Long distance	
Performance group	Women	Men	Women	Men
Q1	58.77 ± 1.47	58.57 ± 1.51	60.92 ± 1.63	60.19 ± 1.55
Q2	58.21 ± 1.91	57.36 ± 1.64	60.18 ± 1.74	59.33 ± 1.95
Q3	57.46 ± 1.96	56.89 ± 1.97	59.41 ± 2.00	58.54 ± 2.04
Q4	56.87 ± 2.59	56.46 ± 2.39	58.06 ± 2.56	57.46 ± 2.59


**FIGURE 2:**
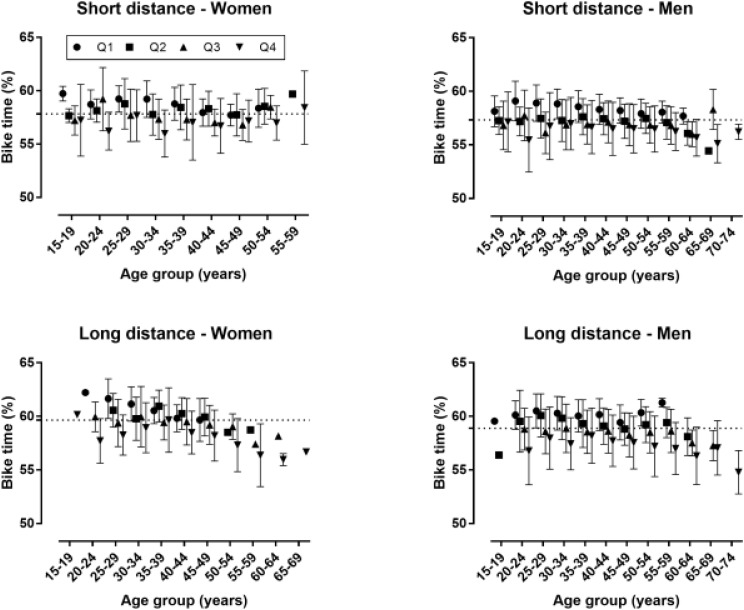
Cycling time (%) by age, performance group, sex, and distance. Q = quartile groups based on race time with Q1 the fastest and Q4 the slowest.

A small age group × performance group interaction on cycling time (%) was observed in the men’s short distance (*p* = 0.001, η_p_^2^ = 0.020) with smaller differences between performance groups in the older than in the younger age groups (Figure 2). No interaction was shown in the women’s short (*p* = 0.397, η_p_^2^ = 0.042) and long distance race (*p* = 0.358, η_p_^2^ = 0.044), and in the men’s long distance race (*p* = 0.256, η_p_^2^ = 0.011).

## Discussion

The main findings of the present study were that (a) men were faster and older, and spent less time (%) in cycling than women in both distances; (b) athletes in older age groups spent less time (%) in cycling than their younger counterparts; (c) athletes in fast performance groups spent more time (%) in cycling than their slower counterparts; and (d) smaller differences between performance groups in the older than in the younger age groups were observed in the men’s short distance.

### Sex Difference in Race Time, Age and Pacing by Distance

The faster race time in men than women was in agreement with previous studies on sex differences in endurance and ultra-endurance sport disciplines, e.g., 50 km ultra-marathon running (Nikolaidis and Knechtle, 2018a) and 6h to 10 days races (Knechtle et al., 2016). The observed sex difference in duathlon performance should be attributed to the physiological correlates of race time in this sport, such as maximal oxygen uptake (Moncada-Jimenez et al., 2009; Berry et al., 2016), where men outscored women. Furthermore, the older age of men than in women confirmed existing research in ultra-endurance running (Nikolaidis and Knechtle, 2018a). An explanation of this sex difference might be the smaller rates of women participation, especially in the older age groups. In addition, the present study was not the first to highlight sex difference in pacing in a multi-sport, as such differences have been already observed in Ironman triathlon (Angehrn et al., 2016).

### Age Difference in Pacing

The less time (%) spent by older age groups in cycling compared to younger age groups indicated that older duathletes were relatively (%) faster in cycling than their younger peers. The observed age-related difference in pacing was in agreement with previous observation on the decline on physiological correlates of performance with aging in triathlon (Wu et al., 2014). Performance in duathlon was characterized by high levels of aerobic capacity (Berry et al., 2016), which in turn might influence fatigue development and the distribution of energy across race. Furthermore, it has been previously observed that performance in duathlon decreased with age (Nikolaidis et al., 2018) and the age-related decline in the duathlon performance was more pronounced in running than in cycling (Rust et al., 2013). The larger effect of aging on running than in cycling has been also shown in comparisons of United States records by age in sport disciplines involving these two locomotion modes (Ransdell et al., 2009). An explanation of the weaker effect of aging on cycling performance than in running might be the larger contribution of technology on the former than the latter locomotion mode (Neptune et al., 2009).

### Combined Effect of Age and Performance on Pacing

An age × performance interaction on pacing was observed only in men for the short distance. It should be reported that, despite no statistically significant, the comparison in the long distance and in women revealed similar effect sizes for the age × performance interaction on pacing, indicating that the lack of statistical significance might be attributed to the small sample sizes in women and longer distance. An interpretation of the smaller differences in pacing among performance groups in the older groups might be that pacing correlated with race time and since differences in race time decreased across age groups, it would be expected that differences in pacing decreased, too. With regards to the role of performance level on pacing, it should be highlighted that fast duathletes were relatively faster in running than in cycling indicating that they redistributed their muscular effort in favor of running. Recently, it was shown that running economy could be improved with running-specific training, whereas cycling-specific training could not enhance cycling economy of endurance athletes (Swinnen et al., 2018). Furthermore, women triathletes could achieve higher cardiorespiratory performance in running than in cycling exercise testing (Snoza et al., 2016). These observations of previous studies might partially explain the relatively better performance of the fastest duathletes in running than in cycling.

### Limitations, Strength and Practical Applications

The findings of the present study might be limited by the specific characteristics of “Powerman Zofingen,” i.e., the separate distances of cycling and running. As differences were observed in pacing between the short and long version of this race, caution would be needed to generalize the findings to other duathlon races differing for total distance as well as for the separate distances of cycling and running. Moreover, it should be highlighted that age groups of either youngest or oldest duathletes were consisted of small sample size. On the other hand, strength of the study was its novelty as it was the first to examine the age × performance interaction on pacing in this sport. Considering the popularity of duathlon, as shown by the increased rates of participation in races of this sport as well as by its use as exercise protocol to study acute physiological responses to combined cycling and running (Berry et al., 2016; Finger et al., 2018), the findings would be of great practical interest for practitioners working with duathletes. Coaches in multi-sports, such as duathlon and triathlon, might train athletes differing for sex, age and performance level; thus, they should acknowledge the variation of pacing between women and men, young and old, fast and slow duathletes, and accordingly, should develop optimal pacing strategies for their athletes. For instance, since fast duathletes were relatively faster in running than in cycling, slow duathletes should be encouraged to cycle slower and run faster.

## Conclusion

In summary, women, young and fast duathletes were relatively slower in cycling than men, old and slow duathletes; that was, old duathletes were relatively faster in cycling than in running. Moreover, there was indication that the difference in pacing among performance groups might be attenuated with aging. Therefore, the distribution of effort between the disciplines of duathlon depended on sex, age and performance level, which was of practical relevance for coaches and fitness trainers.

## Author Contributions

PN performed the statistical analyses and drafted the manuscript. HC, RR, and TR helped in drafting the manuscript. EV collected all the data. BK designed the study and helped in drafting the manuscript. All authors have read and approved the final manuscript.

## Conflict of Interest Statement

The authors declare that the research was conducted in the absence of any commercial or financial relationships that could be construed as a potential conflict of interest.
